# Predicting Pneumothorax and Hemorrhage After CT-Guided Lung Biopsy: Role of Lesion Size, Depth and Their Interaction

**DOI:** 10.3390/jcm14238269

**Published:** 2025-11-21

**Authors:** Rosa Alba Pugliesi, Andreas H. Mahnken, Nour Maalouf, Jonas Apitzsch

**Affiliations:** 1 Section of Radiology, Department of Biomedicine, Neuroscience and Advanced Diagnostics (BiND), University of Palermo, Via del Vespro 129, 90127 Palermo, Italy; 2 Department of Diagnostic and Interventional Radiology, University Hospital of Marburg, 35043 Marburg, Germany; 3 Department of Radiology and Nuclear Medicine, Tübingen University Hospital, 72076 Tübingen, Germany; 4 Department of Radiology and Nuclear Medicine, Helios Hospital Pforzheim, 75175 Pforzheim, Germany

**Keywords:** CT-guided lung biopsy, pulmonary hemorrhage, pneumothorax, lesion size, complication risk, predictive modeling

## Abstract

**Background/Objectives:** CT-guided transthoracic lung biopsy is essential for diagnosing pulmonary lesions but poses risks, chiefly pulmonary hemorrhage and pneumothorax. Both complications can prolong hospitalization or require chest drainage. Emerging evidence suggests that hemorrhage along the biopsy tract may influence pneumothorax risk. This study aimed to evaluate lesion characteristics, tract hemorrhage, and their interaction as predictors of complications, as well as to explore predictive modeling approaches. **Methods:** In this retrospective single-center study, 118 patients (median age 69 years; 55.9% male) underwent CT-TTLB between January 2020 and April 2025. Multivariable logistic regression with Firth correction was used to identify predictors of hemorrhage and pneumothorax, including a lesion depth–hemorrhage interaction. Model discrimination was assessed via bootstrap-corrected ROC analysis. CART analysis defined lesion size thresholds associated with hemorrhage risk. Random Forest and XGBoost models were applied for exploratory comparison. **Results:** Hemorrhage occurred in 29.7% and pneumothorax in 22.0% of cases, with overlap in seven. Larger lesions were less likely to bleed (OR 0.96 per mm, *p* = 0.002), while deeper lesions increased pneumothorax risk (OR 1.06 per mm, *p* = 0.007). The depth–hemorrhage interaction suggested a possible mitigating effect of bleeding in deeper lesions; however, this was an exploratory observation and did not reach conventional statistical significance (*p* = 0.078). **Conclusions:** Limited tract hemorrhage may partially reduce pneumothorax risk in deeper lesions, but this finding is exploratory and requires prospective validation. Lesion size remains the primary determinant of bleeding, and CART-derived thresholds may inform individualized procedural planning and risk stratification.

## 1. Introduction

CT-guided transthoracic lung biopsy (CT-TTLB) is a minimally invasive, well-established procedure for obtaining histopathological diagnoses in patients with pulmonary masses or nodules. It is extremely precise, particularly when utilizing core-needle techniques, and it continues to be indispensable for the assessment of suspected malignancies and inflammatory lung processes [1]. Nevertheless, CT-TTLB is associated with inherent hazards, the most prevalent of which are pneumothorax (PTX) and pulmonary hemorrhage. The incidence of PTX can vary from 15% to over 40%, depending on patient-related factors and procedural details [2,3]. Both complications carry practical clinical implications: PTX may necessitate chest tube drainage, prolong hospitalization, or delay oncologic therapy, while hemorrhage can result in hemoptysis or transient respiratory compromise [4].

These complications, which were previously considered adverse events, have recently been reassessed in light of their potential interrelationship. Mild or trace hemorrhages may not be indicative of genuine complications, but rather of anticipated procedural outcomes [5]. Experimental evidence suggests that parenchymal bleeding could act as a natural sealant, mitigating air leakage and lowering PTX risk—a concept supported by studies showing that tract embolization with gelatin sponge reduces both PTX and hemorrhage [6,7]. Nevertheless, the exact interaction between lesion characteristics, bleeding, and PTX remains incompletely understood.

Procedural standardization has proven effective in decreasing complication rates. Optimization of needle angulation within a defined “safe zone,” coupled with operator training, reduced PTX incidence from 71.1% to 21.8% across three learning phases [8]. Our institution previously identified this “safe zone” for the needle–pleura angle (between 80° and 100°) in a cohort of 52 patients, showing that deviations greater than 10° from perpendicularity markedly increased PTX risk [9]. All biopsies included in the present, expanded cohort (*n* = 118) were subsequently performed within this validated safe angulation range, ensuring procedural consistency and adherence to established best practice. Building upon this standardized technical foundation, the current study shifts the focus toward the role of pulmonary hemorrhage as a potential modifier of PTX risk. In a subsequent institutional study of 112 patients, we also found that while PTX occurred in nearly 44% of biopsies, most cases were asymptomatic and required no intervention, supporting the safety of symptom-based rather than routine post-biopsy imaging [10]. Collectively, these findings emphasize how both procedural refinement and critical reassessment of post-procedural management can improve patient safety.

The cohort analyzed here partially overlaps with previous single-center investigations from our group that examined needle–pleura angle optimization, technical standardization, and imaging follow-up protocols using smaller subsets of patients [8,9,10]. Those earlier studies provided essential procedural context but did not address the relationship between pulmonary hemorrhage and PTX. The current expanded analysis (*N* = 118) integrates and extends that work by specifically evaluating hemorrhage as a potential modifier of PTX risk.

Taken together, prior and current investigations underscore that CT-TTLB complications have multifactorial origins. Lesion size and depth have shown inconsistent associations with PTX, and their potential interaction with hemorrhage remains poorly characterized [1,3]. CART analysis and other advanced multivariable modeling and classification methods may be employed to establish clinically meaningful thresholds that will help guide procedural planning and mitigate risk.

Consequently, the aim of this study was to assess the incidence and predictors of PTX and pulmonary hemorrhage in a modern CT-TTLB cohort. The study focused on the interacting determinants of complication risk, more specifically hemorrhage, size, and depth of the lesion. A secondary objective was to establish size-based cutoff values that could be employed to create secure procedural strategies.

## 2. Materials and Methods

This retrospective single-center cohort study included 118 patients (66 males, 52 females; median age 69, range 49–90) who underwent CT-guided transthoracic lung biopsy (CT-TTLB) between 9 January 2020, and 4 April 2025. The institutional review board gave its ethical approval (Ref: F-2021-038), and the procedures followed the Declaration of Helsinki. All interventions were performed by a single board-certified interventional radiologist with 18 years of experience.

### 2.1. Inclusion and Exclusion Criteria

Patients were recalled from the institutional radiology database by procedural codes for CT-guided biopsies. The inclusion criteria considered an intrapulmonary lesion that was a candidate for CT-guided biopsy and imaging after the procedure within 7 days. Exclusion criteria included lesions <4 mm, INR > 1.5, prior chest tube placement, aborted or technically failed procedures, inability to follow breath-hold instructions, and absence of post-biopsy imaging. Lesions amenable to bronchoscopy or located in the pleura or mediastinum were not considered in this study.

### 2.2. Biopsy Protocol

Each biopsy was performed using an 18 G semi-automatic Tru-Cut needle with a notched inner stylet and sliding outer sheath, inserted through a 17 G coaxial trocar (Möller Medical GmbH, Fulda, Germany) under a 128-slice Siemens Somatom Definition Edge CT (Forchheim, Germany). Procedures were carried out with patients in either the prone or supine position. CT images were obtained during end-expiratory breath-hold with a slice thickness of 2 mm and without intravenous contrast. Subcutaneous infiltration of 1% mepivacaine provided local anesthesia. Biopsy specimens were immediately fixed in formalin and submitted for histopathological analysis. Each intervention involved a single pleural passage, with no prophylactic autologous blood patches applied. Number of biopsy samples ranged from 2–4 per lesion depending on lesion size and operator judgment. Needle corrections were performed as needed; deeper or smaller lesions required more adjustments. Dwell time was consistent with standard practice and trajectory length was determined by lesion depth plus estimated subpleural distance. These details enhance reproducibility and clarify procedural nuances.

### 2.3. Measurement Yield

Lesion characteristics (maximum diameter, depth from pleura to lesion center, and segmental location) were recorded. Lesions were stratified by size (≤10 mm, 11–20 mm, >20 mm) and depth quartiles. The presence of emphysematous changes along the biopsy needle path was evaluated on CT images, rather than relying solely on clinical COPD diagnosis. Patients were classified as having emphysematous changes if ≥1 segment along the tract showed evidence of emphysema.

The primary outcome was PTX, defined as any intrapleural air on post-biopsy CT.

Delayed PTX within 24–72 h was captured if clinically suspected. Symptomatic PTX required respiratory symptoms and intervention. Chest drain placement followed institutional criteria and was adjudicated by the performing radiologist. Standardized symptom monitoring included chest pain, dyspnea, tachycardia, cough, and auscultation at multiple time points (immediate post-procedure, pre-transfer, 1 h, 4 h, evening, and morning ward rounds).

Secondary outcomes included pulmonary hemorrhage defined as any new perilesional or parenchymal hyperdensity along the biopsy tract visible on immediate post-procedure CT. Hemoptysis was recorded if clinically evident. Follow-up imaging within 7 days was only performed if clinically indicated. Minor versus major hemorrhage analysis was not performed due to small number of patients.

### 2.4. Statistical Analysis

Continuous variables were summarized as means ± standard deviation or medians with interquartile ranges; categorical variables were reported as frequencies and percentages. Bivariate comparisons used Chi-square or Fisher’s exact tests for categorical variables, and Mann–Whitney U or *t*-tests for continuous data, depending on distribution.

To identify independent predictors of PTX, a multivariable logistic regression model was developed with PTX as the outcome. The key independent variable was pulmonary hemorrhage. Covariates included lesion size and depth, lesion location, COPD status, age, sex, and patient position. An interaction term between hemorrhage and lesion depth was included to test for effect modification. Multicollinearity was assessed using variance inflation factors (VIF), and model performance via AUC-ROC. Sensitivity analyses excluded extreme values (outliers) of lesion size and depth. A *p*-value < 0.05 was considered statistically significant. Analyses were performed using R (version 4.5.0). There were no missing data for the primary analytical variables (lesion size, depth, hemorrhage, and PTX). All cases with incomplete auxiliary clinical information (*n* = 2) were excluded from the relevant analyses, ensuring complete-case modeling.

To evaluate whether machine learning models could provide exploratory comparison of predictive performance, Random Forest and XGBoost algorithms were trained using the same clinical variables as in the logistic regression. Model hyperparameters were optimized using grid search with cross-validation. Performance metrics included area under the curve (AUC), balanced accuracy, sensitivity, specificity, and Cohen’s kappa coefficient. Comparative performance was assessed, and results were presented in Supplementary Table S1. All machine learning analyses were conducted using the caret and xgboost packages in R. To assess the robustness of the logistic regression model, we performed internal validation using non-parametric bootstrapping with 1000 resamples. For each resample, the AUC was calculated, and the optimism-corrected AUC was derived as a measure of model discrimination.

## 3. Results

**Figure 1:**
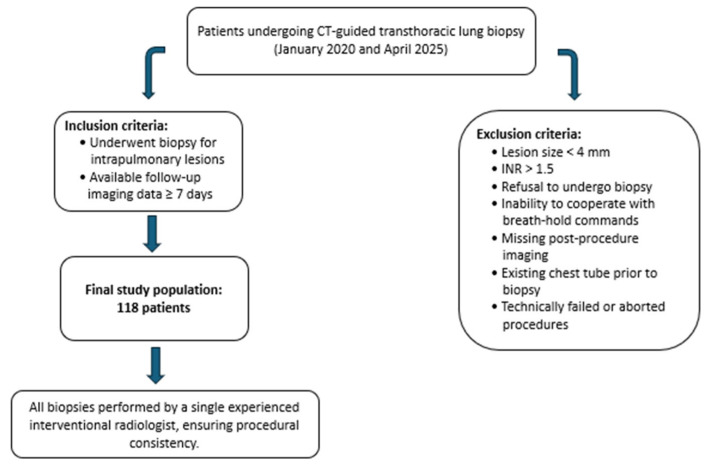
Flowchart illustrating inclusion and exclusion criteria for patients undergoing CT-guided transthoracic lung biopsy. All procedures were performed by a single experienced interventional radiologist.

The data from a total of 118 patients that underwent CT-guided lung biopsy was collected (Figure 1).

Procedures were performed with patients in the supine position in 65 cases (55.1%) and prone in 53 cases (44.9%). COPD was present in 39 patients (33.1%). The mean lesion size was 38.6 ± 26.6 mm (range 2.6–115 mm), and the mean lesion depth was 25.1 ± 20.7 mm. Lesions were most frequently located in the right upper lobe (32.2%) and right lower lobe (29.7%).

PTX occurred in 26 patients (22.0%). Rates were higher for smaller lesions (≤10 mm: 29.4%) and deeper lesions (21.5–41 mm: 30.0%) compared to larger or more superficial lesions. Patients with COPD had a slightly lower PTX rate than those without COPD (17.9% vs. 24.1%). Chest tube placement was required in 7 cases (5.9%), and hemorrhage occurred in 35 patients (29.7%), with an overlap of both complications in 7 patients (Table 1).

The logistic regression model incorporating lesion size, lesion depth, and hemorrhage demonstrated an optimism-corrected AUC of 0.71 (bootstrap, 1000 resamples), indicating fair discriminative performance for predicting pneumothorax risk.

The multivariable logistic regression results are summarized in Table 2.

Lesion depth independently increased PTX risk, while larger lesions were protective: each 1 mm increase in depth raised odds by ~6%, and each 1 mm increase in size lowered odds by ~4% (these percentages are derived from the ORs: depth OR = 1.06 → (1.06 − 1) × 100 ≈ 6%; size OR = 0.96 → (1 − 0.96) × 100 ≈ 4%), highlighting lesion morphology’s procedural relevance.

Hemorrhage alone was not significant, but the hemorrhage × depth interaction approached significance (*p* = 0.078), suggesting a possible mitigating effect. Model performance details and sensitivity analyses are provided in Supplementary Table S3.

### 3.1. Histological Outcomes

Histopathology was successfully obtained in 116 out of 118 patients (98.3%), while 2 cases (1.7%) were considered as non-diagnostic due to either insufficient material or the absence of any tumor cells. In diagnostic cases, malignancies accounted for 77.6% of the cohort. The most frequent malignant subtype was adenocarcinoma, which occurred in 39.8% of patients, followed by squamous cell carcinoma (14.4%), non-small-cell lung cancer not otherwise specified (NSCLC NOS) in 11.9%, and pulmonary metastases in 5.9%. Neuroendocrine tumors, as evidenced by carcinoids and atypical variants, accounted for 5.1% of diagnoses. Other malignancies, such as entities comprising sarcoma and fibrous tumors, accounted for 7.6%. Benign and inflammatory lesions were present in 13.6% of the cases, including organized pneumonia, chronic inflammation, fibrosis, granulomatous disease, and hamartomas. The distribution of histological findings is illustrated in Supplementary Figure S1.

### 3.2. Predictors of Pneumothorax

**Figure 2:**
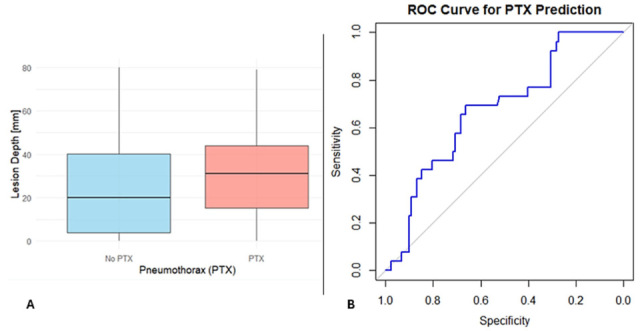
Lesion Depth and Pneumothorax Prediction. (**A**) Boxplot showing greater lesion depth in patients with pneumothorax compared to those without. (**B**) ROC curve for lesion size and depth predicting PTX, with an AUC of 0.675, sensitivity 69.2%, and specificity 66.3%. The blue line represents the model’s performance, while the grey diagonal line indicates random guessing.

Multivariable logistic regression analysis identified greater lesion depth as a significant predictor of PTX (β = 0.056, *p* = 0.007), whereas larger lesion size was protective (β = –0.042, *p* = 0.007). As shown in Figure 2A, patients who developed PTX had greater lesion depths compared to those who did not.

Hemorrhage alone was not predictive (*p* = 0.639), but the interaction between lesion depth and hemorrhage approached significance (β = −0.061, *p* = 0.078) and should be interpreted as an exploratory, hypothesis-generating observation. Any significant associations were observed for biopsy position, COPD status, or lesion location (all *p* > 0.05).

To assess the overall predictive power of lesion features, a ROC analysis was performed using lesion depth and size. The combined model showed fair discriminatory ability, with an AUC of 0.675, sensitivity of 69.2%, and specificity of 66.3% (Figure 2B). These findings highlight that lesion depth and size are strong predictors but that the low AUC indicates that other clinical or procedural factors may also play a role in PTX risk.

**Figure 3:**
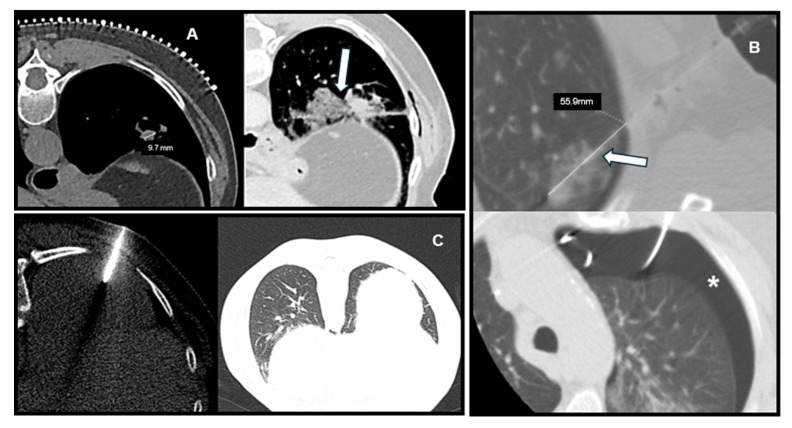
Representative CT Images of Lesion Characteristics and Post-Biopsy Complications. (**A**) Small subpleural lesion (<30 mm) with focal parenchymal hemorrhage after biopsy (arrow). (**B**) Deep-seated lesion (>40 mm to pleura) with long intrapulmonary needle path (arrow) and resultant pneumothorax (star). (**C**) Superficial lesion with no pneumothorax or hemorrhage, illustrating low-risk profile.

Representative CT images illustrating the relationship between lesion characteristics and post-biopsy complications are shown in Figure 3, including a deep lesion with PTX, a small lesion with hemorrhage, and a superficial lesion without complications.

**Figure 4:**
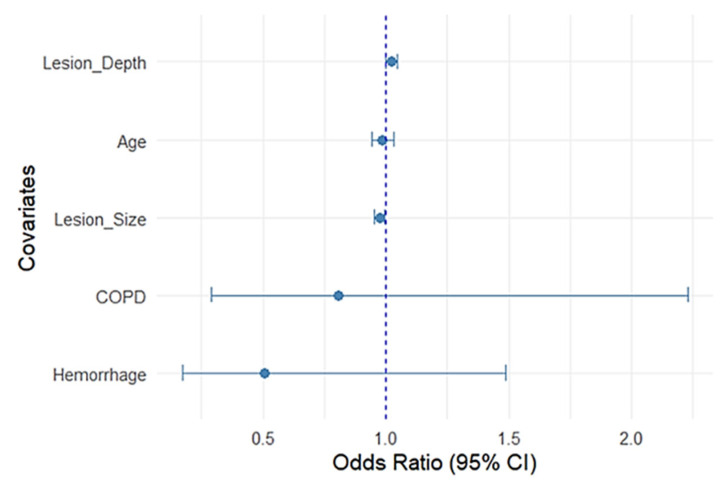
Predictors Effects on Pneumothorax Risk. Forest plot of adjusted odds ratios from multivariable logistic regression. Lesion depth increased risk; larger lesion size was protective. Error bars represent the 95% confidence interval (CI). The dashed vertical line at an odds ratio of 1 indicates no effect on pneumothorax risk.

To further illustrate the relative contribution of each variable, a forest plot was generated from the multivariable regression model (Figure 4).

**Figure 5:**
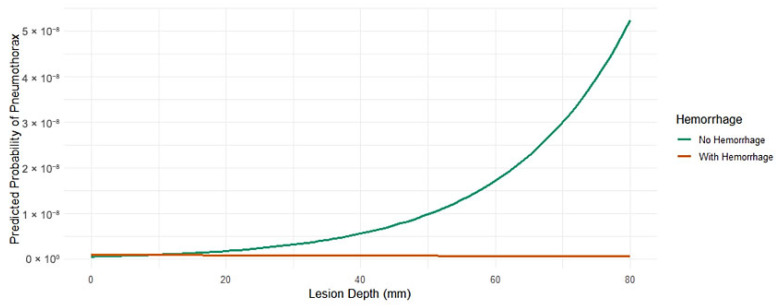
Interaction Effects on Pneumothorax Risk. Interaction plot showing predicted pneumothorax probability versus lesion depth, with (red) and without (green) hemorrhage.

Lesion depth remained positively associated with PTX, while lesion size was inversely related; in other words, the smaller the lesion, the more likely a PTX occurred (Figure 5).

Hemorrhage, COPD, and age were not significant. Narrow confidence intervals for the lesion parameters reflect precise estimates compared with wider intervals seen with comorbidity variables, reflecting greater uncertainty.

Further evaluation of clinical utility using decision curve analysis demonstrated added net benefit over default strategies across relevant threshold probabilities. Additional model diagnostics, including decision curve analysis (Supplementary Figures S2–S4), calibration (Supplementary Figure S5), and a predictive nomogram (Supplementary Figure S6) are presented in the Supplementary Material.

### 3.3. Comparison of Predictive Models Using Machine Learning

To explore whether machine learning can better perform predictions than logistic regression, we trained models using Random Forest and XGBoost (R 4.5.0) on the same clinical predictors. While logistic regression achieved the best AUC (0.709), the best-balanced accuracy (0.569) and greatest agreement (kappa = 0.145) were achieved using XGBoost achieved the best-balanced accuracy and agreement, but overall performance remained comparable and not superior to logistic regression. Random Forest gave the best sensitivity (0.926) but at the cost of low specificity. Supplementary Table S1 presents a comparative performance summary of Random Forest, logistic regression, and XGBoost models.

### 3.4. Predictors of Hemorrhage

In a separate regression model evaluating predictors of hemorrhage, lesion size was the only variable that reached statistical significance. Specifically, larger lesions were less prone to hemorrhage (β = −0.039, *p* = 0.002). No significant associations were observed for lesion depth, biopsy position, emphysematous changes, or lesion location. A possible explanation is that larger lesions are technically easier to target, reducing the need for needle adjustments during the procedure. Needle corrections may not always necessitate repeated pleural puncture, but they could elevate the risk of parenchymal injury and subsequent hemorrhage. These findings show how important the shape of the lesion, especially its size, is in determining the risk of bleeding after a biopsy. Decision curve analysis demonstrated a consistent net clinical benefit of the hemorrhage prediction model across a range of relevant thresholds (Supplementary Figure S3).

### 3.5. Interaction Between Lesion Depth and Hemorrhage

Despite the absence of a statistically significant main effect of hemorrhage on PTX (*p* = 0.639), the interaction term approached statistical significance (β = −0.061, *p* = 0.078), hinting at a potential moderating effect on the relationship between lesion depth and PTX risk. These interaction effects were further explored by using a stratified subgroup analysis, with findings detailed in Supplementary Table S2.

### 3.6. CART Analysis

**Figure 6:**
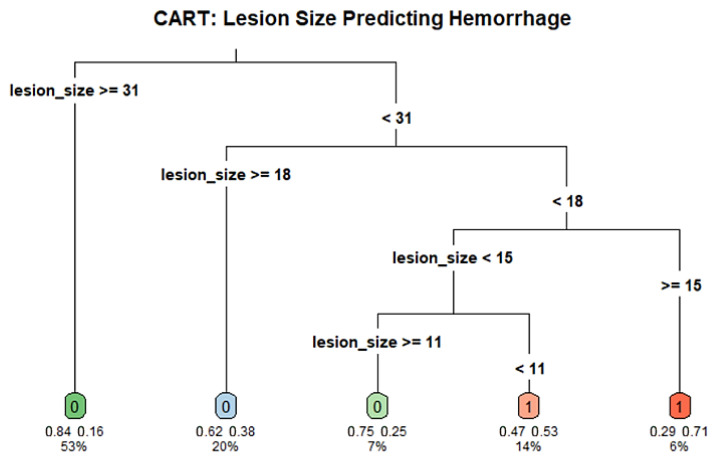
CART Predicting Hemorrhage Based on Lesion Size. Classification and Regression Tree (CART) model illustrating lesion size thresholds associated with post-biopsy hemorrhage risk. Each node displays the predicted class (0 = no hemorrhage; 1 = hemorrhage), class probabilities, and percentage of patients in that node. The root node splits at 31 mm, with additional splits at 18 mm, 15 mm, and 11 mm further stratifying risk. Lesions smaller than 11 mm were associated with the highest hemorrhage probability (53%). Box colors indicate risk levels, with blue-green denoting lower risk and red-orange indicating higher hemorrhage likelihood.

To identify potential cutoff values for the association lesion size and post-biopsy hemorrhage, we conducted a CART analysis using lesion size as the sole predictor of hemorrhage. The model included 118 patients and used a minimum terminal node size of 5 (minbucket = 5) and a complexity parameter of 0.01. The resulting tree split the cohort at a number of thresholds, the most important first split at a lesion diameter of 30.5 mm. Individuals with lesions ≥30.5 mm were at lower risk of hemorrhage (16.1%) compared to those with lesions <30.5 mm (44.6%). The decision tree further stratified the risk at multiple lesion diameter thresholds, with key splits occurring around 10.5 mm and 17.5 mm, that so indicates a nonlinear relationship between size and PTX risk. While the global rate of misclassification was negligible, the tree suggests that smaller lesion diameter could be associated with greater risk of hemorrhage. The decision nodes were then divided in subsequent branches, which could, however, be indicative of overfitting. These findings may inform future threshold selection for procedural planning but are at risk of cautious interpretation due to the limited sample number and lack of external validation. The full decision tree structure and hemorrhage risk thresholds are illustrated in Figure 6.

## 4. Discussion

This study evaluated predictors of PTX and pulmonary hemorrhage following CT-guided transthoracic lung biopsy in a contemporary cohort, with an emphasis on lesion characteristics and interactions.

Our results confirm that lesion depth is a risk factor for PTX, while increased lesion size is associated with protection, consistent with prior studies [11,12]. The potential moderating effect of hemorrhage on the relationship between lesion depth and PTX risk provides additional nuance. This interaction was not statistically significant, but the continuous tendency implies that small tract bleeding may partially protect against PTX by closing alveolar defects [13]. This exploratory and hypothesis-generating observation needs broader cohort validation. Post hoc power analysis assessed the study’s power at ~67%, suggesting that the small number of events may hinder detection of subtle interaction effects and model stability. Internal bootstrap validation suggested reasonable internal consistency, but external multicenter validation is required to confirm these findings.

Emphysema/COPD was assessed on CT along the intended needle tract and included as a covariate in the models; however, it did not attain statistical significance. It is acknowledged that these variables may induce potential confounding, and the impact of these variables on the risk of PTX may be more clearly defined in larger studies. The findings of this cohort were primarily influenced by lesion-related factors, as all biopsies were administered using validated needle–pleura angulation parameters (80–100° “safe zone”), which reduced procedural variability. The observation that tract hemorrhage may serve as a physiologic “sealant” aligns with prior experimental work [9], suggesting that small parenchymal bleeds could limit the spread of air along alveolar planes [14]. While mechanistically plausible [15,16], this effect requires confirmation in prospective studies or controlled experimental models.

Among the strengths of the study is the fact that it was a single-center cohort with standardized biopsy technique by a senior interventional radiologist, which reduced procedural heterogeneity. The use of a previously validated safe-angle protocol adds methodological rigor and strengthens the internal consistency of results across the overlapping institutional series. The detailed lesion characterization and use of multivariable modeling and CART analyses allowed a comprehensive assessment of risk factors and potential thresholds for clinical practice. The use of Firth logistic regression, a conservative method of reducing small-sample bias, also contributes to the validity of our findings, particularly in the analysis of rare outcomes. However, there are some limitations that need to be considered. First, the retrospective design itself is prone to selection bias and causal inference is limited. The sample size, while moderate, limited the power to identify subtle effects, especially in subgroup analyses. In particular, the limited number of patients who required chest drainage after PTX reduced the ability to adequately examine predictors of this clinically important endpoint. A post hoc power analysis estimated the study’s power to be approximately 67%, details of which are provided in Supplementary Table S3. Attempts at modeling drainage risk were compromised by statistical instability and overfitting with the low number of events, and CART analyses for drainage were not informative. Therefore, we chose to report these findings for completeness but not to feature them in the general conclusions, in line with suggestions in the literature for larger multicenter cohorts to clarify etiologies of severe PTX requiring intervention [17].

Furthermore, certain patient-related risk factors such as emphysema or aberrant pulmonary function did not reach statistical significance in our model, whereas prior studies have reported discordant findings for these variables [18,19]. Institutional protocol and biopsy method heterogeneity may also affect complication rates and generalizability. Exclusion of lesions accessible via bronchoscopy and pleural-based masses targets the study but decreases scope in intrapulmonary nodules. Notably, our findings may not be generalizable to peripheral lesions accessible via other modalities, like robotic-assisted bronchoscopy, whose popularity has grown in recent years [13,20].

Our findings align with previous meta-analyses confirming PTX and hemorrhage as common complications of CT-guided biopsy but suggest that lesion size and depth are key procedural variables modifiable in planning [21]. The exploratory observation of a possible protective effect of hemorrhage warrants prospective evaluation with potential influence on post-biopsy observation and management protocols [12,22].

Integrating lesion morphometry into pre-biopsy risk assessment algorithms may improve clinical decision-making, especially in patients with borderline performance status or comorbid pulmonary disease.

In particular, smaller and deeper lesions may warrant alternative approaches such as real-time imaging guidance, robotic navigation, or adjunctive techniques to minimize complication risk. This discriminative lesion-based planning could potentially minimize risk with preserved diagnostic yield [16].

Emerging studies have begun incorporating artificial intelligence (AI) and radiomics to predict biopsy-related complications using high-dimensional imaging features beyond what is visible to the human eye [15,23,24]. These models have shown promising early results but require further validation and clinical integration [25]. Our findings could complement such AI-based tools by providing interpretable, biologically grounded predictors (e.g., size, depth, hemorrhage interaction), which may improve model transparency and clinician trust. Future directions should explore hybrid approaches combining radiomics, clinical risk scores, and procedural data for real-time decision support.

Additionally, inspection of the operator’s technique (such as needle path optimization, breath-hold guidance, coaxial systems) and the use of adjunctive maneuvers (such as sealant plug deployment, saline infusion or blood patch) could also reduce complication rates and would merit study in concentrated prospective trials.

Internal validation through bootstrapping indicated that the predictive model maintained stable performance, supporting the internal consistency of the results. By ensuring a standardized biopsy approach within the validated safe-angle parameters, this study isolates lesion-related factors as key determinants of complication risk. However, the absence of external validation remains an important limitation, and future research should aim to replicate these findings across different institutions and operator settings. Larger, prospective cohorts could help establish generalizable risk thresholds and validate whether pulmonary hemorrhage indeed mitigates PTX occurrence. Ultimately, integrating lesion-based risk profiling with procedural standardization may optimize patient safety and diagnostic yield in CT-guided lung biopsy.

## 5. Conclusions

These findings indicate that pulmonary hemorrhage during CT-TTLB may diminish the risk of PTX, particularly in deep lesions, whereas lesion size is the most significant predictor of hemorrhage. Identifying bleeding as a possible protective factor may guide procedural choices. Lesion size criteria developed using CART provide a pragmatic instrument for risk categorization. Future prospective, multicenter trials are essential to validate these findings and further refine personalized techniques that improve safety during CT-TTLB.

## Figures and Tables

**Table 1:** Patient Characteristics, Lesion Features, and Procedural Complications Following CT-Guided Lung Biopsy. Summary of baseline demographic data, lesion characteristics, and post-biopsy complication rates in 118 patients undergoing CT-guided transthoracic lung biopsy (CT-TTLB). Lesion depth was defined as the distance from the pleural surface to lesion center. COPD was defined based on clinical and imaging criteria.

Variable	Value
Total patients	118
Sex, *n* (%)	Male: 66 (55.9%), Female: 52 (44.1%)
Median age (years)	69 (range: 49–90)
COPD status, *n* (%)	Present: 39 (33.1%)
Biopsy position, *n* (%)	Supine: 65 (55.1%), Prone: 53 (44.9%)
Mean lesion size (mm ± SD)	38.6 ± 26.6
Mean lesion depth (mm ± SD)	25.1 ± 20.7
Lesion location by lobe, *n* (%):	
− Right Upper Lobe (RUL) − Right Middle Lobe (RML) − Right Lower Lobe (RLL) − Left Upper Lobe (LUL) − Left Lower Lobe (LLL)	38 (32.2%) 15 (12.7%) 35 (29.7%) 16 (13.6%) 14 (11.9%)
Pneumothorax	26/118 (22.0%)
By lesion size	
− ≤10 mm − 11–20 mm − >20 mm	5/17 (29.4%) 6/23 (26.1%) 15/78 (19.2%)
By lesion depth	
− 0–7.25 mm − 7.25–21.5 mm − 21.5–41 mm − 41–80 mm	3/30 (10.0%) 7/29 (24.1%) 9/30 (30.0%) 7/29 (24.1%)
By COPD status	
− No COPD − With COPD	19/79 (24.1%) 7/39 (17.9%)
Chest tube placement	7/118 (5.9%)
Pulmonary hemorrhage	35/118 (29.7%)
Overlap: Pneumothorax and Hemorrhage	7/118 (5.9%)

**Table 2:** Multivariable Logistic Regression for Predictors of Pneumothorax Following CT-Guided Lung Biopsy.

Predictor	Adjusted OR	95% CI	*p*-Value
Lesion depth (per mm)	**1.06**	1.02–1.11	**0.007**
Lesion size (per mm)	**0.96**	0.94–0.99	**0.002**
Pulmonary hemorrhage (yes vs. no)	0.85	0.41–1.77	0.639
Hemorrhage × Lesion depth (interaction)	0.94	0.88–1.01	0.078
COPD (yes vs. no)	0.75	0.28–1.99	0.56
Age (per year)	0.99	0.95–1.03	0.57
Patient position (prone vs. supine)	1.12	0.48–2.63	0.80

Multivariable logistic regression model with Firth bias correction evaluating predictors of pneumothorax. Bold values indicate statistical significance (*p* < 0.05). Clinically, each 1 mm increase in lesion depth raises pneumothorax odds by ~6%, while each 1 mm increase in lesion size lowers odds by ~4%, highlighting lesion morphology’s procedural impact. CI = confidence interval; OR = odds ratio.

## Data Availability

The data presented in this study are openly available in Pugliesi, Rosa Alba (2025). CT-Guided Lung Biopsy Interactions. figshare. Dataset. https://doi.org/10.6084/m9.figshare.29847860.v1.

## Supplementary Materials

The following supporting information can be downloaded at https://www.mdpi.com/article/10.3390/jcm14238269/s1, Supplementary Figure S1: Distribution of Histological Diagnoses Among Biopsied Lung Lesions. Bar chart showing the distribution of histological diagnoses with percentages. NSCLC NOS indicates non-small-cell lung carcinoma not otherwise specified. The Benign/Inflammatory group comprises non-neoplastic findings, including organized pneumonia, chronic inflammatory changes, and pulmonary fibrosis. Supplementary Figure S2: Decision curve analysis. The curve is showing the standardized net benefit of using the prediction model for pneumothorax across a range of threshold probabilities (0 to 0.5). The red curve represents the model’s net benefit. The black horizontal line (“None”) indicates the net benefit of treating no patients, while the gray diagonal line (“All”) represents the net benefit of treating all patients. The area where the model’s curve lies above both lines indicates a clinical advantage of using the model to guide decision-making. Supplementary Figure S3: Decision curve analysis for predicting the need for drainage following pneumothorax. The model (red line) shows higher net benefit than default strategies within low-to-moderate risk thresholds, suggesting clinical utility in identifying patients who may require chest tube placement. Supplementary Figure S4: Decision curve analysis for predicting hemorrhage risk. The hemorrhage model (red line) outperformed both the “treat-all” and “treat-none” strategies across the majority of threshold probabilities, highlighting its potential application in tailoring post-procedural monitoring. Supplementary Figure S5: Calibration curve of the logistic regression model predicting pneumothorax occurrence. The dashed line represents perfect calibration (ideal model). The dotted line shows the apparent calibration based on the sample data, and the solid line represents the bias-corrected calibration estimated using 1000 bootstrap resamples. The closer the bias-corrected line is to the ideal, the better the model’s calibration. Supplementary Figure S6: Nomogram for predicting the risk of post-interventional pneumothorax. The nomogram is based on a multivariable logistic regression model incorporating Lesion Depth (mm), Lesion Size (mm), Age (years), and the presence of COPD. To estimate an individual patient’s risk of pneumothorax, locate each variable on its respective axis and draw a vertical line to the “Points” axis to determine how many points each predictor contributes. Sum all points and locate the total on the “Total Points” axis. Draw a vertical line downward to determine the corresponding probability of pneumothorax on the “Risk of Pneumothorax” axis. Supplementary Table S1: Comparative performance of three prediction models for pneumothorax (Logistic Regression, Random Forest, XGBoost). Supplementary Table S2: Subgroup analysis of pneumothorax predictors stratified by COPD status. Supplementary Table S3: Sensitivity analysis excluding lesions with zero depth or size (*N* = 67). Exclusion of zero-valued lesions led to decreased model discrimination (AUC: 0.571), although direction and magnitude of effect estimates remained largely consistent with the primary model. This suggests these outliers values contribute to a meaningful predictive signal, and their inclusion may enhance model stability.
