# Supplementary material for: Predicting Pneumothorax and Hemorrhage After CT-Guided Lung Biopsy: Role of Lesion Size, Depth and Their Interaction
Source: J Clin Med. 2025 Nov 21;14(23):8269. doi: 10.3390/jcm14238269 (PMC12693312; doi:10.3390/jcm14238269)
Supplement: Supplementary file 1 [file jcm-14-08269-s001.zip › jcm-3994760-supplementary.pdf]

Figure S1

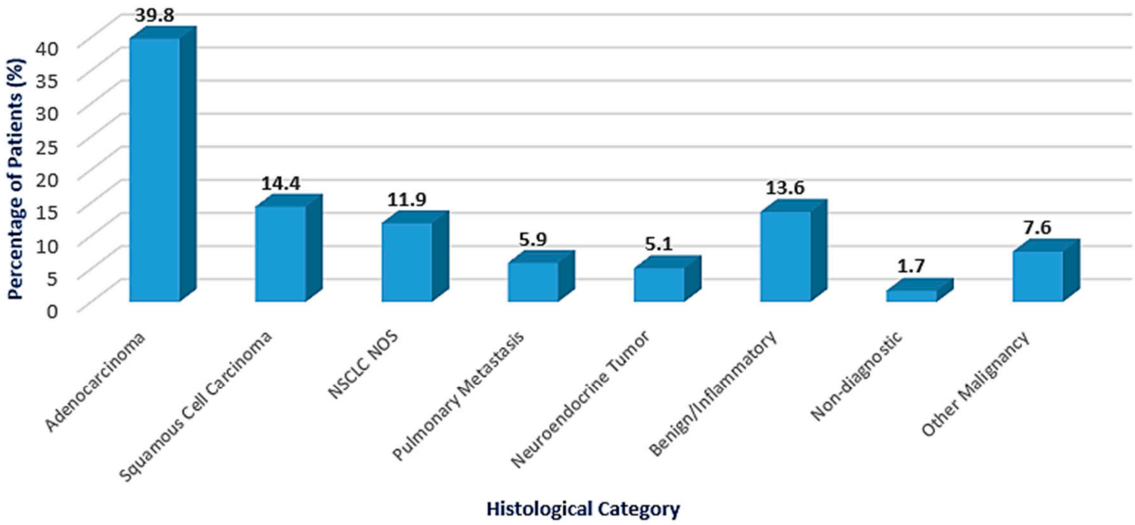

Supplementary Figure S1. Distribution of Histological Diagnoses Among Biopsied Lung Lesions. Bar chart showing the distribution of histological diagnoses with percentages. NSCLC NOS indicates non-small-cell lung carcinoma not otherwise specified. The Benign/Inflammatory group comprises non-neoplastic findings, including organized pneumonia, chronic inflammatory changes, and pulmonary fibrosis.

Figures S2–S4

Clinical Utility – Decision Curve Analysis

To assess the clinical utility of the pneumothorax prediction model, we performed a Decision Curve Analysis (DCA) using standardized net benefit as the primary metric. As shown in Supplementary Figure S2, the model consistently demonstrated greater net benefit than the "treat-all" and "treat-none" strategies across a clinically relevant range of threshold probabilities (0.05–0.35). This indicates that implementing the model in clinical practice could improve patient outcomes by better identifying patients who may benefit from preventive interventions based on individualized risk.

Supplementary Figures S2–S4 show decision curve analyses for predicting pneumothorax, hemorrhage, and drainage requirements. Across relevant threshold probabilities (0 to 0.5), all three models demonstrated improved net benefit compared to default strategies, supporting their potential value in guiding risk-informed clinical decisions.

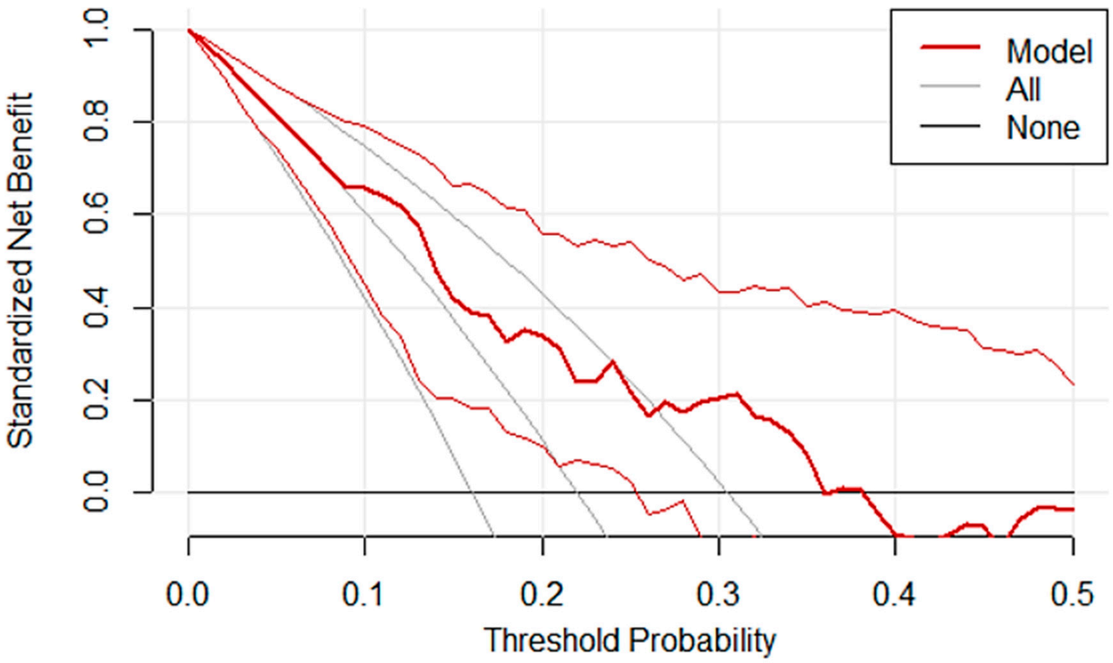

Supplementary Figure S2: Decision curve analysis. The curve is showing the standardized net benefit of using the prediction model for pneumothorax across a range of threshold probabilities (0 to 0.5). The red curve represents the model's net benefit. The black horizontal line ("None") indicates the net benefit of treating no patients, while the gray diagonal line ("All") represents the net benefit of treating all patients. The area where the model's curve lies above both lines indicates a clinical advantage of using the model to guide decision-making.

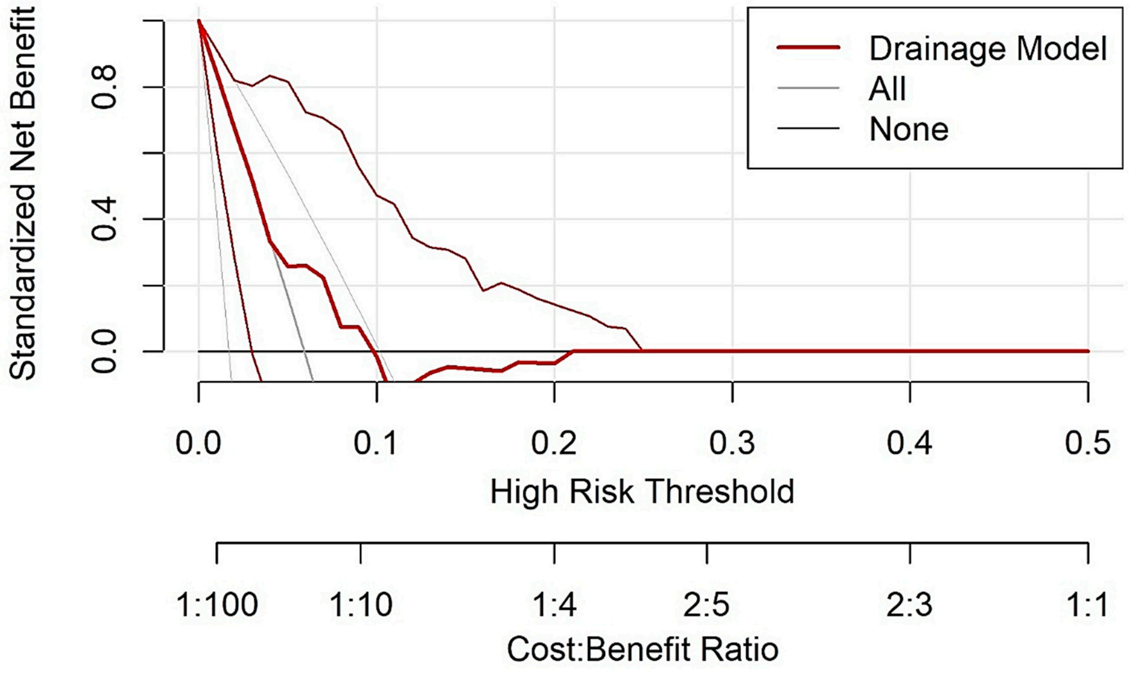

Supplementary Figure S3: Decision curve analysis for predicting the need for drainage following pneumothorax. The model (red line) shows higher net benefit than default strategies within low-to-moderate risk thresholds, suggesting clinical utility in identifying patients who may require chest tube placement.

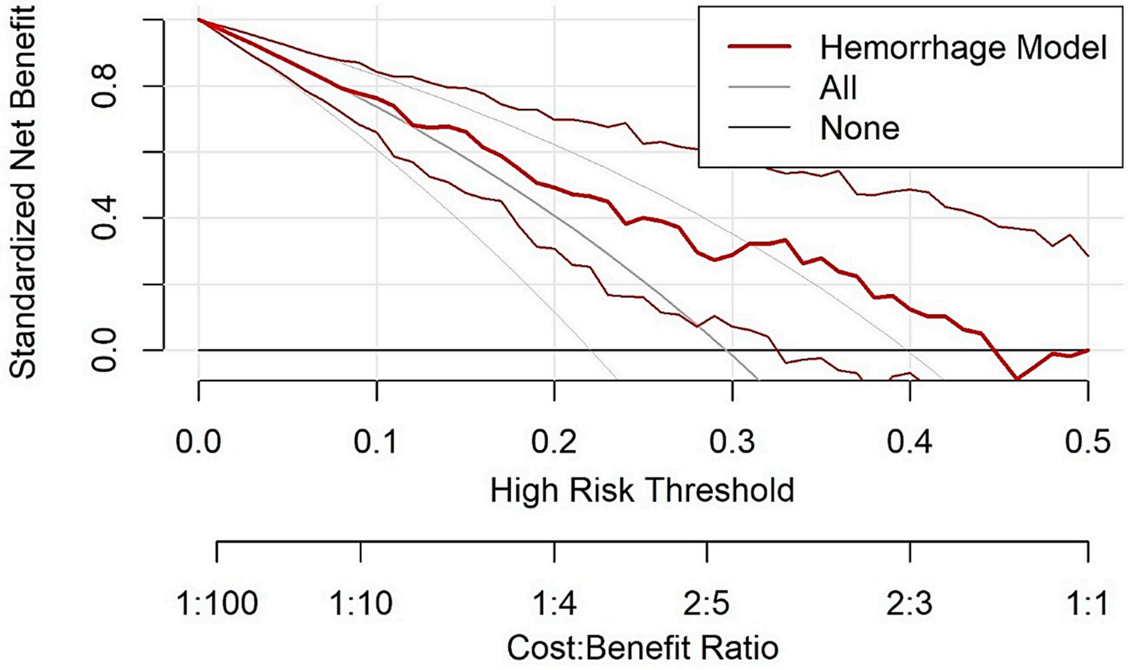

Supplementary Figure S4: Decision curve analysis for predicting hemorrhage risk. The hemorrhage model (red line) outperformed both the "treat-all" and "treat-none" strategies across the majority of threshold probabilities, highlighting its potential application in tailoring post-procedural monitoring.

Figure S5

Model Calibration

To evaluate model calibration, we plotted a calibration curve using 1,000 bootstrap resamples. The bias-corrected line closely approximated the ideal 45-degree line, indicating good concordance between predicted and observed pneumothorax probabilities. The mean absolute error was 0.035, suggesting minimal miscalibration.

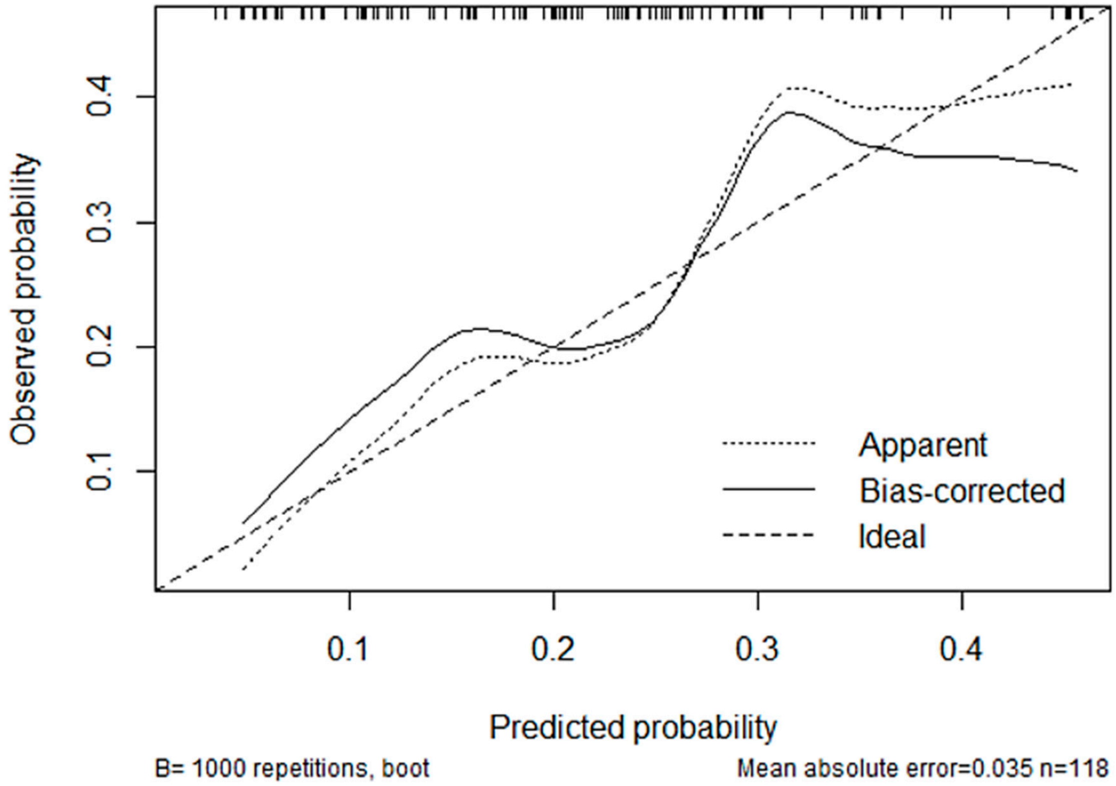

Supplementary Figure S5:

Calibration curve of the logistic regression model predicting pneumothorax occurrence. The dashed line represents perfect calibration (ideal model). The dotted line shows the apparent calibration based on the sample data, and the solid line represents the bias-corrected calibration estimated using 1000 bootstrap resamples. The closer the bias-corrected line is to the ideal, the better the model's calibration.

Figure S6

Nomogram Section

To enhance clinical usability, we developed a nomogram based on the final multivariable logistic regression model for pneumothorax prediction. The model incorporated Lesion Depth, Lesion Size, Age, and COPD status as independent predictors. The nomogram allows clinicians to estimate an individual patient's risk by assigning variable-specific point values and summing them to derive a predicted probability. This tool could facilitate real-time, personalized risk communication during procedural planning.

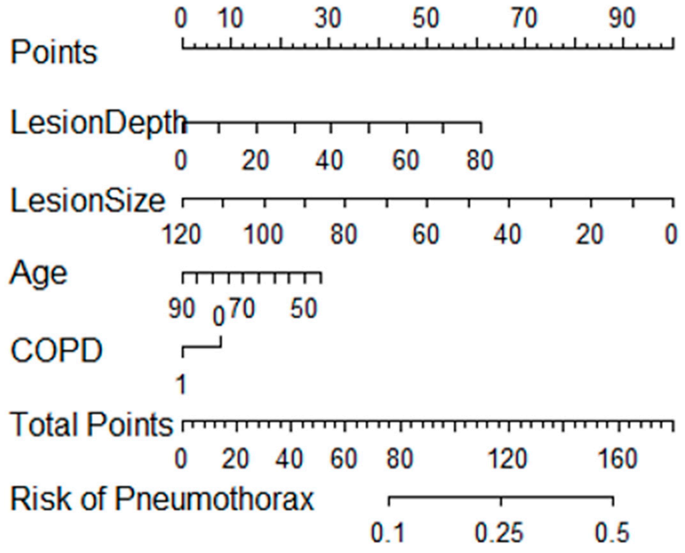

Supplementary Figure S6. Nomogram for predicting the risk of post-interventional pneumothorax. The nomogram is based on a multivariable logistic regression model incorporating Lesion Depth (mm), Lesion Size (mm), Age (years), and the presence of COPD. To estimate an individual patient's risk of pneumothorax, locate each variable on its respective axis and draw a vertical line to the "Points" axis to determine how many points each predictor contributes. Sum all points and locate the total on the "Total Points" axis. Draw a vertical line downward to determine the corresponding probability of pneumothorax on the "Risk of Pneumothorax" axis.

Supplementary Table S1.

**Metric**	**Logistic Regression**	**Random Forest**	**XGBoost**
AUC	0.709	0.688	0.698
Accuracy	0.735	0.765	0.735
Sensitivity (Class 0)	0.889	**0.926**	0.852
Specificity (Class 1)	0.143	0.143	**0.286**
Balanced Accuracy	0.516	0.534	**0.569**
Kappa	0.038	0.087	**0.145**

Supplementary Table S1. Comparative performance of three prediction models for pneumothorax (Logistic Regression, Random Forest, XGBoost).

While logistic regression achieved the highest AUC (0.709), the XGBoost model had superior balanced accuracy and kappa, indicating better overall calibration between sensitivity and specificity. Random Forest achieved the highest sensitivity for negative cases but at the expense of low specificity.

Subgroup Analysis by COPD Status

Stratified logistic regression was conducted to assess whether lesion-related predictors differed by COPD status. Among patients with COPD (n = 39), lesion size trended toward significance with good discrimination (AUC = 0.75). In the non-COPD group (n = 79), lesion depth approached statistical significance (p = 0.068), although the model had more modest performance (AUC = 0.669). These findings suggest that risk predictors may differ by underlying lung disease, warranting further validation.

**Variable**	**OR (95% CI) – COPD+**	**p-value**	**OR (95% CI) – COPD–**	**p-value**
Lesion Depth	1.01 (0.95 – 1.03)	0.768	1.02 (1.00 – 1.06)	0.068
Lesion Size	0.96 (0.91 – 1.00)	0.070	0.98 (0.95 – 1.01)	0.183
Age	0.96 (0.87 – 1.05)	0.459	0.99 (0.94 – 1.05)	0.564
**AUC**	**0.75**	—	**0.669**	—

Supplementary Table S2. Subgroup analysis of pneumothorax predictors stratified by COPD status.

Post Hoc Power Analysis

A post hoc power analysis was conducted to assess whether the sample size (N = 118) provided adequate power to detect a clinically meaningful association between lesion depth and pneumothorax. Based on the observed odds ratio (OR = 1.02, 95% CI: 1.00–1.04), and assuming a baseline event rate of 20% versus 40% in exposed individuals, the estimated power was ~67% at $\alpha = 0.05$. Although slightly below the conventional 80% threshold, this level is acceptable given the exploratory nature of the study.

Sensitivity Analysis

**Predictor**	**OR (Original)**	**OR (Sensitivity)**	**AUC (Original)**	**AUC (Sensitivity)**
Lesion Depth	1.02 (1.00–1.04)	1.00 (0.97–1.03)	0.709	0.571
Lesion Size	0.98 (0.96–1.00)	0.99 (0.96–1.02)		
Age	0.98 (0.94–1.03)	0.98 (0.93–1.03)		
COPD	0.81 (0.30–2.21)	0.52 (0.14–1.87)		

Note: ORs shown as adjusted values with 95% confidence intervals.

Supplementary Table S3. Sensitivity analysis excluding lesions with zero depth or size (N = 67). Exclusion of zero-valued lesions led to decreased model discrimination (AUC: 0.571), although direction and magnitude of effect estimates remained largely consistent with the primary model. This suggests these outliers values contribute to a meaningful predictive signal, and their inclusion may enhance model stability.
